# Developmental changes in gaze patterns in response to radial optic flow in toddlerhood and childhood

**DOI:** 10.1038/s41598-022-15730-5

**Published:** 2022-07-07

**Authors:** Nobu Shirai, Tomoko Imura

**Affiliations:** 1 Department of Psychology, Faculty of Humanities, Niigata University, 2-8050 Ikarashi Nishi-Ku, Niigata, 950-2181 Japan; 2 Department of Psychology, Faculty of Integrated Arts and Social Sciences, Japan Women’s University, 2-8-1 Mejirodai Bunkyo-ku, Tokyo, 112-8681 Japan; 3 Present Address: Department of Psychology, College of Contemporary Psychology, Rikkyo University, 1-2-26 Kitano Niiza-shi, Saitama, 352-8558 Japan

**Keywords:** Psychology, Human behaviour, Sensorimotor processing, Sensory processing, Visual system

## Abstract

A large field visual motion pattern (optic flow) with a radial pattern provides a compelling perception of self-motion; a radially expanding/contracting optic flow generates the perception of forward/backward locomotion. Moreover, the focus of a radial optic flow, particularly an expansive flow, is an important visual cue to perceive and control the heading direction during human locomotion. Previous research has shown that human gaze patterns have an “expansion bias”: a tendency to be more attracted to the focus of expansive flow than to the focus of contractive flow. We investigated the development of the expansion bias in children (*N* = 240, 1–12 years) and adults (*N* = 20). Most children aged ≥ 5 years and adults showed a significant tendency to shift their gaze to the focus of an expansive flow, whereas the youngest group (1-year-old children) showed a significant but opposing tendency; their gaze was more attracted to the focus of contractive flow than to the focus of expansive flow. The relationship between the developmental change from the “contraction bias” in early toddlerhood to the expansion bias in the later developmental stages and possible factors (e.g., global visual motion processing abilities and locomotor experiences) are discussed.

## Introduction

Optic flow is a large-field visual motion pattern that appears in human sight during movement and is a strong cue to perceive and control a person’s locomotor action^1–5^. For instance, when a person walks toward the frontal direction, radial expansive flow typically occupies the visual field. In this situation, the focus of radial optic flow that appears in the human visual field is an important cue to perceive and control the person’s heading direction; the position of the focus of the radial flow on the visual field is always consistent with the person’s heading direction in the environment at any particular moment. However, there are several controversies regarding the use of radial optic flow focus in the perception/control of heading direction^6,7^.

The human visual system has a unique ability to detect the focus of radial optic flow, which may be relevant to heading perception/action. Niemann et al.^8^ demonstrated that naive adults tend to gaze around the focus of the radial optic flow when they passively view radial flow stimuli. They also indicated that the gaze pattern is more attuned to expansion flows, which represent forward locomotion, than to contraction flows, which represent backward locomotion. The “expansion bias” in gaze responses to radial optic flow suggests that the human visual system may be tuned to detect visual cues, the focus of radial expansion flow, to perceive/control the heading direction during forward locomotion.

Many studies have shown that a robust ability to detect radial optic flow is present in humans, including very young infants at a few months of age (cf. a review by Shirai & Yamaguchi^9^). However, several studies have reported that the ability to detect the focus of radial flow remains immature during the first few years of life. Gilmore and colleagues^10,11^ reported that although 3–5-month-old infants discriminated between radial flows that had different focus positions, their discrimination threshold for the position of the focus of the radial optic flow (> 20°) was much higher than the discrimination threshold of adults (< 2°). More recently, Shirai and Imura^12^ investigated whether infants aged between 4 and 18 months (divided into five groups: 4- to 6-month-olds, 7- to 9-month-olds, 10- to 12-month-olds, 13- to 15-month-olds, and 16- to 18-month-olds) and adults show similar expansion bias in gaze patterns toward radial expansion/contraction flows, compared with adult participants in the study by Niemann et al. (1999). Shirai and Imura reported that adult observers indicated an expansion bias (looked significantly longer around the focus of expansion flow, compared with contraction flow), similar to the results reported by Niemann et al. (1999). However, Shirai and Imura found markedly different results in infants: younger groups aged 4–6 and 7–9 months exhibited a significant “contraction bias” (looked significantly longer around the focus of contraction flow, compared with expansion flow), and the groups > 10 months of age exhibited no significant difference in gaze responses to the focus of radial flow between expansion and contraction flows. Moreover, Shirai and Imura (2016) showed that total time looking at the focus of radial flow was shorter in infants than in adults, while latency to detect the focus of radial flow was longer in infants. These findings suggest that although some basic abilities to detect the focus of radial flow patterns emerge within the first half-year of life, these abilities are immature and thus not comparable with adult patterns.

Because the expansion bias in gaze responses to radial flow patterns observed in adults may be related to perception/control of the heading direction, this expansion bias may be acquired through locomotor experience after birth. Although Shirai and Imura (2016) reported that the tendency in gaze behavior to the focus of radial flow was not significantly different between infants who had locomotor experience and infants who had no locomotor experience, the acquisition of the expansion bias in gaze behaviors to the focus of radial optic flow may occur during later stages of life than infancy/toddlerhood. Therefore, the main aim of the present study was to investigate when the adult-like expansion bias in gaze responses to the focus of radial flow emerges in human life. We investigated the development of gaze behaviors to the focus of radial expansion/contraction flows in children between 1 and 12 years of age (compared with adults) using the experimental procedure described by Shirai and Imura (2016).

In the current study, we adopted two measures, namely, looking time and latency of gaze pattern shift to the focus of radial flow, to estimate expansion bias in keeping with an adaptive perspective on locomotor behaviors. Looking time was adopted as a main measure because a longer time looking at the focus of the radial flow pattern seems to reflect the provision of stable control for locomotor action. Because the heading direction during locomotor action would be frequently updated in a natural situation (e.g., walking down a winding road, avoiding an obstacle on the locomotion path, etc.), our visual system should continuously monitor the position of the focus of radial flow showing our heading direction to appropriately control our locomotor action. The ability to hold our gaze on the focus of the radial flow pattern would seem an aid for monitoring the position of the focus of radial flow. Moreover, in addition to our previous study^12^, recent studies by other research groups have shown that spontaneous and sustainable gaze responses to the focus of radial optic flow are observed in a variety of participants/subjects (e.g., human infants^12^, adults^12–14^, and nonhuman primates [macaque and marmoset]^14^). Thus, a longer time looking at the focus of radial flow can serve as a common and useful index of better performance in detecting the focus of radial flow. Latency of the first gaze response toward the focus of radial flow would also indicate an ability relevant to locomotion control; rapid detection of the focus of radial flow would result in faster recognition of the momentary direction of locomotion, particularly for the starting phase of locomotion.

## Method

### Ethics statement

This study was approved by the Ethics Committee for Psychological Research of Niigata University and was conducted in accordance with the Declaration of Helsinki. Written informed consent was obtained from all participants or their parents, as appropriate.

### Participants

The final sample was composed of 20 adults and 240 1- to 12-year-old children. The children were divided into 12 age groups. Detailed information on the age groups is summarized in Table 1. Nineteen additional children participated in the experiment but were excluded from the final sample because of eye tracker calibration failure (n = 9), inattention to visual stimuli (n = 6), and experimenter errors (n = 4). Moreover, two more adults were excluded from the final sample because of eye tracker calibration failure.

**Table 1:** Details of participant ages in each of the age groups.

Age group	*N* (female:male)	Mean age in years ± 1*SD*	Age range in years
1-year-old	20 (8:12)	1.63 ± 0.29	1.03–1.97
2-year-old	20 (11:9)	2.56 ± 0.32	2.01–2.99
3-year-old	20 (11:9)	3.45 ± 0.31	3.05–3.93
4-year-old	20 (10:10)	4.52 ± 0.23	4.05–4.99
5-year-old	20 (9:11)	5.50 ± 0.30	5.04–5.99
6-year-old	20 (13:7)	6.47 ± 0.31	6.02–6.99
7-year-old	20 (11:9)	7.58 ± 0.34	7.06–7.98
8-year-old	20 (9:11)	8.58 ± 0.29	8.08–8.94
9-year-old	20 (9:11)	9.35 ± 0.28	9.03–9.96
10-year-old	20 (9:11)	10.48 ± 0.32	10.04–10.91
11-year-old	20 (7:13)	11.42 ± 0.31	11.01–11.96
12-year-old	20 (11:9)	12.48 ± 0.31	12.02–12.98
Adults	20 (13:7)	19.84 ± 0.64	18.94–21.63

### Apparatus and stimulus

Because all apparatus and stimuli were identical to those used by Shirai and Imura (2016)^12^, the following descriptions thereof are partially based on those in that study^12^. In an experimental booth, a 21-inch cathode ray tube (CRT) monitor (Nanao, FlexScan T966, resolution = 1,024 × 768 pixels, refresh rate = 60 Hz) was placed in front of each participant and used to display visual stimuli. A pair of loudspeakers was placed behind the CRT monitor to produce “beeping” sounds at the beginning of each experimental trial to attract the participant’s attention to the monitor. An eye tracker (Tobii Technology, Tobii X120, sampling rate = 60 Hz) was set at the bottom of the monitor to record the participant’s gaze patterns during the experiments. Tobii Studio software (Tobii Technology) running on a personal computer (Dell, Precision T1700, CPU: Xeon E3-1240 v3 3.40 GHz, RAM: 16 GB, video card: Nvidia Quadro K600 1 GB) was used to control stimuli presentation, record gaze patterns, and perform the data analysis. A liquid crystal display (LCD) computer monitor, connected to a personal computer, was placed outside the experimental booth to allow an experimenter to monitor the eye-tracking data and visual stimuli In real-time during the experiment. A small video camera attached just below the CRT monitor was connected to a small video monitor near the experimenter, enabling the experimenter to monitor the looking behaviors of participants (and their caregivers) in real time.

**Figure 1:**
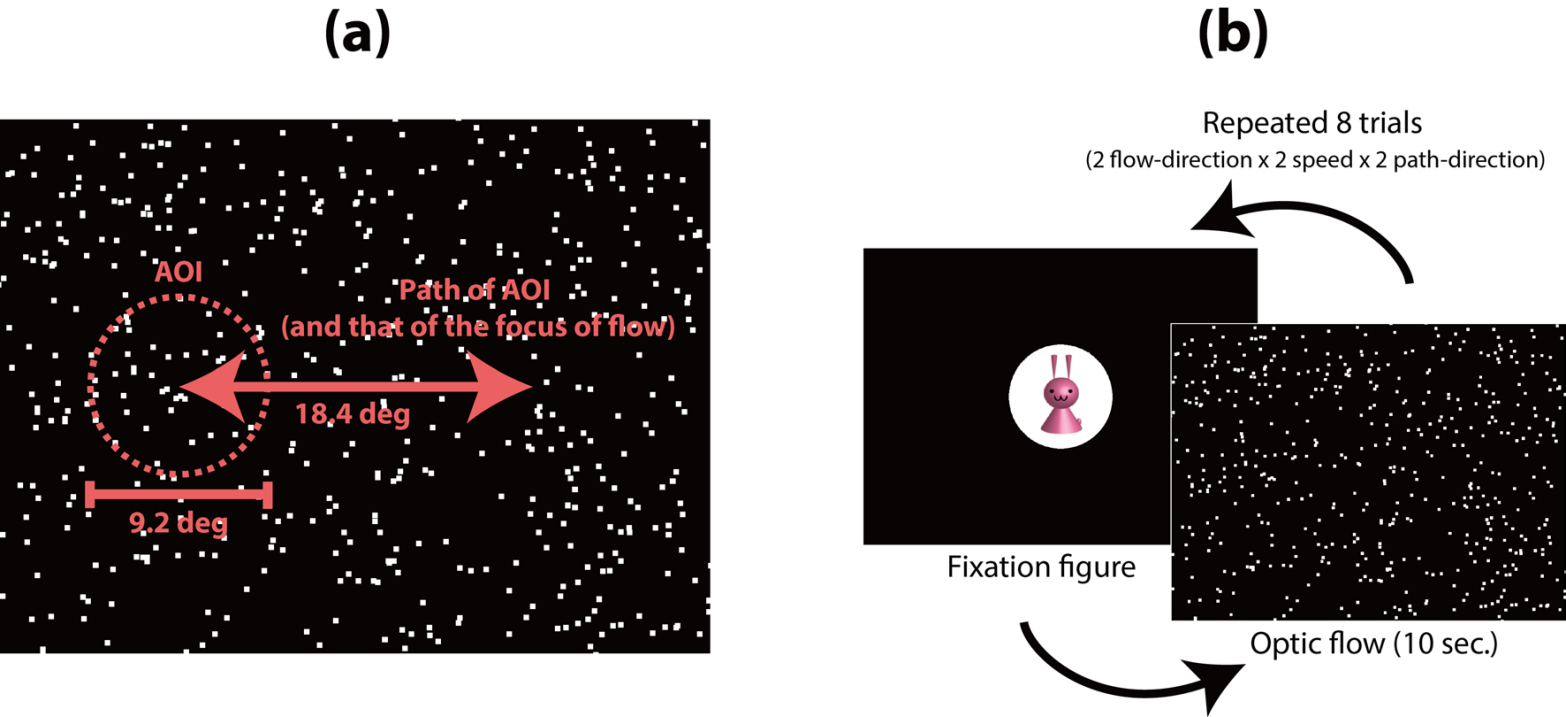
(**a**) Schematic diagram of the motion paths of the area of interest (AOI) and the focus of the radial flow pattern. (**b**) Flowchart of the experimental procedure. These figures were adopted from Shirai & Imura (2016, *Scientific Reports*, https://doi.org/10.1038/srep34734), in accordance with a Creative Commons Attribution 4.0 International License. The apparatus, visual stimuli, and experimental procedures of the current study were identical to the method used by Shirai & Imura (2016).

All visual stimuli were movies of radial flow patterns in an uncompressed audio video interleave (AVI) format (resolution = 1024 × 768 pixels, frame rate = 60 frames/s, duration = 10 s; all movie files used in the current study have been uploaded to “https://nyu.databrary.org/volume/252”). Each flow pattern was composed of 500 bright moving dots (luminance = 85.0 cd/m^2^, size = 0.3 deg^2^) presented on a dark background (luminance = 9.1 cd/m^2^, size = 35.3° × 26.4°). Each dot moved along a radial pattern (either expansion or contraction) at intervals of 33.3 ms; thus, the flow pattern appeared as a 30 frames/s animation. The speed of each dot (deg/s) in each animation frame was calculated by multiplying the constant (0.33 under the low-speed condition, 0.66 under the high-speed condition) by the eccentricity (in visual degrees) of the dot’s position from the focus of radial optic flow. Thus, the dot speed was zero at the focus of radial optic flow and increased linearly toward the periphery. Mean dot speeds across the whole flow pattern were approximately 5.8 deg/s and 11.6 deg/s under the low and high-speed conditions, respectively. The lifetime of each dot was 10 frames (333 ms). Dots randomly re-appeared on the background at the end of their lifetime or if they moved out of the background. A contraction flow movie was generated by playing an expansion movie in reverse. AVI movies of expansion and contraction flows were composed of exactly the same set of video frames but presented in the opposite order. Thus, we first generated a frame set (a total of 300 images) for an expansion flow pattern and combined them in the original order (from frame no. 1 to frame no. 300) to make an AVI movie of an expansion flow (the source code for the program used to generate the frame set is available at “https://doi.org/10.6084/m9.figshare.20057333.v1”). Then, we combined the same frame set in the reverse order (from frame no. 300 to frame no. 1) to make a movie of a contraction flow. Thus, there were no differences in visual properties across the whole presentation period between the expansion and contraction flow movies, with the exception of the flow direction of the dots. The focus of radial optic flow was positioned at either the left or right side of the background at the start of each movie. The focus of radial optic flow was initially positioned at 9.2 deg to the left or right of the screen center, and then moved by approximately 3.7 deg/s toward the opposite side and then back to its original position, over a 10 s period (Fig. 1a). Benchmark movies were adopted to compare the abilities of participants in different age groups to control eye movement. Each benchmark movie was composed of a single white moving square (luminance = 85.0 cd/m^2^, size = 1.9 deg^2^) presented in the background. The motion trajectory of the square was identical to the motion trajectory of the focus of radial optic flow in the optic flow movie.

All visual stimuli used in Shirai and Imura (2016) and the current study are available at “https://nyu.databrary.org/volume/252”.

### Procedure

Because we followed the experimental procedure of Shirai and Imura (2016)^12^ in the current study, the following description of the procedure is partially based on that study^12^.

Each participant sat on a chair in front of the CRT monitor without any supportive equipment, such as a head or chin rest. Participants who were young children (mainly 1- and 2-year-olds) sat on their caregivers’ laps and were held in front of the CRT monitor. The viewing distance was approximately 65 cm. Before the experiment, a calibration procedure for the eye tracker was conducted using a calibration program with five calibration points built into Tobii Studio software.

Before they began the experiment, participants were told to “Please watch the computer monitor as you would watch the TV in your home.” No other specific instructions were provided. In experiments involving young children held by their caregivers, the caregivers were instructed before each experiment to close their eyes during the trials, thus ensuring that they would be naive to the identities of the stimuli. The experimenter monitored the participants’ and caregivers’ looking behaviors via the video monitor. If the caregivers opened their eyes before they completed the experiment, the experimenter asked them to close their eyes in the intervals between trials during the experiment. Each experimental trial began with a fixation figure (a colorful cartoon character displayed on a white circular window [diameter = 9.2°]) appearing at the center of the CRT monitor accompanied by beeping sounds. When an experimenter (observing the LCD monitor) confirmed that the participant gazed at the fixation figure, the experimenter initiated the presentation of a radial flow movie. The duration of each flow movie was 10 s. Each participant completed eight experimental trials: expansion and contraction × low- and high-speed conditions × 2 repetitions (2 motion paths for the focus of radial optic flow: right/left/right or left/right/left). The order of the trials was randomized. After the eight experimental trials, each participant viewed a pair of benchmark movies (right/left/right or left/right/left). The order of the two benchmark movies was counterbalanced across participants.

### Experimental conditions and data analyses

Two within-participants independent factors were used: flow direction (expansion and contraction) and dot speed (low and high). Thus, there were four experimental conditions (expansion with low speed, expansion with high speed, contraction with low speed, and contraction with high speed) per participant in the current study. Furthermore, participant age group was used as a between-participant independent factor.

A circular area of interest (AOI: subtended 9.2°) was arbitrarily defined on the focus of radial optic flow (or the center of a white square during a benchmark movie), and the motion trajectory of the AOI was identical to the motion trajectory of the focus of radial optic flow (or the white square) in each movie (Fig. 1b). Two measures regarding the participant’s gaze patterns toward the AOI were adopted: looking time and latency. Looking time was the total looking time at the AOI in each trial. Latency was the time required for the participant's gaze to reach the AOI following fixation on the attention-getting figure. If a participant did not look inside the AOI at all during a particular trial, the latency in the trial was regarded as 10 s (equal to trial duration). Looking time and latency over eight trials (the four experimental conditions × 2 trials) for participants in each age group were used as dependent measures.

Another AOI was established: AOIW (AOI for the whole area of the visual stimulus). The shape and size of the AOIW were equivalent to the background (35.3° × 26.4°), such that the AOIW fully covered the whole area of each visual stimulus. This AOIW and the benchmark movies (see Procedure for details) were used in additional analyses to examine possible artifacts in the results.

We used the lmerTest package (version 3.1.3) for R software (version 4.2.0) to conduct analyses of variance (ANOVAs) with a linear mixed-effects model. We used the lsmeans R package (version 2.30.0) for post hoc analyses of the ANOVA results (pairwise comparisons of least-squares means with adjustment for degrees of freedom via the Kenward–Roger method).

The raw data for the current study are available at “https://nyu.databrary.org/volume/1317”.

## Results and discussion

### Summary of individual results for looking time Fig. 2)

**Figure 2:**
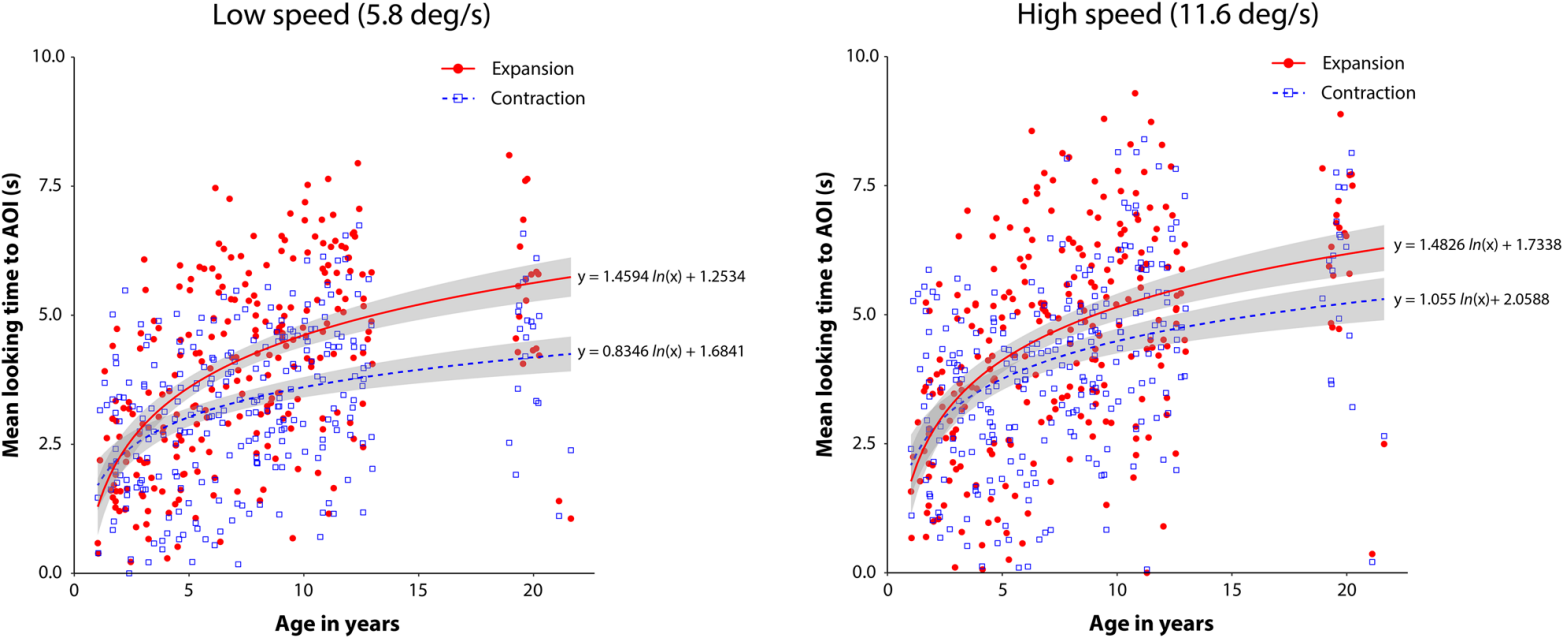
Individual mean looking time under each experimental condition. The left and right panels summarize the results for the low- and high-speed conditions, respectively. In each panel, the vertical axis shows the mean time looking at the AOI, and the horizontal axis shows the age in years of the individual participants. Red dots and blue squares indicate individual mean looking times for the expansion and contraction flow patterns, respectively. Solid red and dotted blue lines represent logarithmic regression curves for the expansion and contraction flow conditions, respectively. Gray stripes indicate 95% confidence intervals. The regression curves and confidence intervals were obtained using the ggplot2 R package (ver. 3.3.5).

Figure 2 shows the mean time looking at the AOI under each of the experimental conditions. Overall, in both the low- (5.8 deg/s) and high-speed (11.6 deg/s) conditions, the mean looking time gradually increased with participant age. Moreover, a pronounced expansion bias, i.e., more time looking at the AOI of expansion than contraction flows, seemed to appear after toddlerhood.

### Main analysis for looking time Fig. 3a)

**Figure 3:**
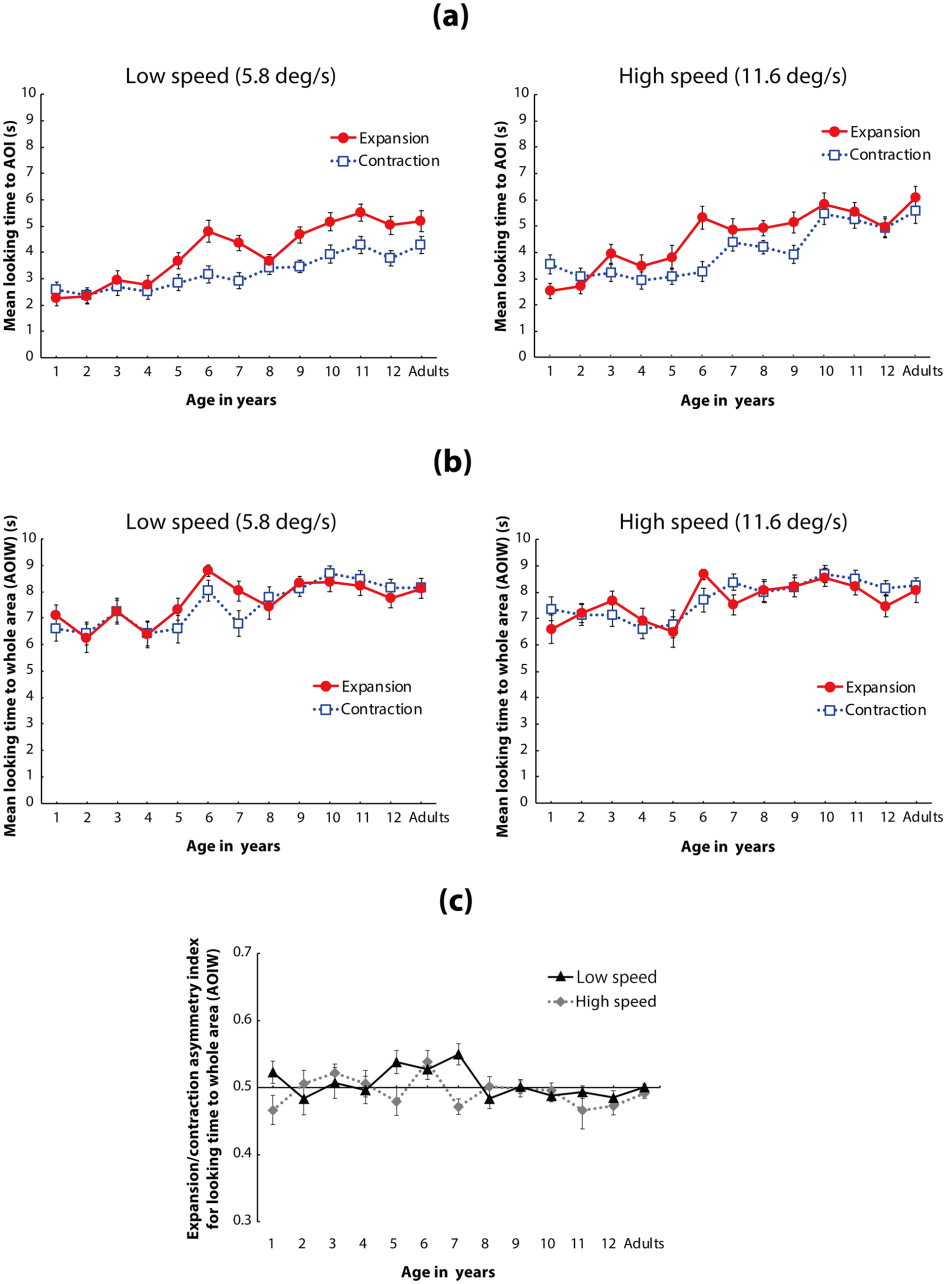
(a) Mean time looking at the AOI of the flow patterns. In each panel, the vertical axis shows the mean time looking at the AOI, and the horizontal axis shows the age group of the participants. Solid red lines with circles represent mean time looking at expansion flow patterns, and dotted lines with open squares represent mean time looking at contraction flow patterns. Left and right panels show the results under low-speed (5.8°/s) and high-speed (11.6°/s) conditions, respectively. Error bars indicate ± 1 *SEM*. (b) Mean time looking at the whole area (AOIW) of the flow patterns. In each panel, the vertical axis represents mean time looking at the AOIW, and the horizontal axis represents the age group of the participants. Solid red lines with circles represent mean time looking at expansion flow patterns, and blue dotted lines with open squares represent mean time looking at contraction flow patterns. Left and right panels show the results under low-speed (5.8°/s) and high-speed (11.6°/s) conditions, respectively. Error bars indicate ± 1 *SEM*. (c) Asymmetry index of looking time for the expansion and contraction flows in the AOIW analysis (vertical axis). The index for each participant was calculated as the total looking time for the expansion flow divided by the total looking time for both the expansion and contraction flows. Mean index values higher and lower than 0.5 indicate looking behaviors biased toward expansion and contraction, respectively. Error bars indicate ± 1 *SEM*.

A three-way analysis of variance (ANOVA) with a linear mixed-effects model (fixed factors: age [13] × flow direction [2] × dot speed [2], random factor: participants) revealed that the main effects of age, flow direction, and dot speed were statistically significant (*F*(12,247) = 12.073, *p* < 0.001, η_*p*_^2^ = 0.37 (95% CI: 0.26, 0.44); *F*(1,247) = 51.470, *p* < 0.001*,* η_*p*_^2^ = 0.17 (95% CI: 0.10, 0.26); and *F*(1,247) = 82.631, *p* < 0.001, η_*p*_^2^ = 0.25 (95% CI: 0.16, 0.34), respectively). The interaction between age and flow direction was also statistically significant (*F*(12,247) = 3.698, *p* < 0.001*,* η_*p*_^2^ = 0.15 (95% CI: 0.05, 0.20), as was that between flow and speed (*F*(1,247) = 3.938, *p* = 0.048*,* η_*p*_^2^ = 0.02 (95% CI: 0.00, 0.06)). The other interactions were not statistically significant (age × speed: *F*(12,247) = 1.349, *p* = 0.192*,* η_*p*_^2^ = 0.06 (95% CI: 0.00, 0.08); age × direction × speed: *F*(12,247) = 1.561, *p* = 0.104*,* η_*p*_^2^ = 0.07 (95% CI: 0.00, 0.10)). The aim of the current study was to examine the development of asymmetric gaze responses toward the focus of a radial optic flow pattern between expansion and contraction flows. Thus, we used the following detailed analyses regarding the interaction between age and flow direction.

The simple main effect of flow direction in each age group was statistically significant in 1-, 5-, 6-, 7-, 9-, 10-, 11-, and 12-year-olds and in adults, but it was not statistically significant in 2-, 3-, 4-, and 8-year-olds (*ps* < 0.05). These results indicate that 1-year-old children spent significantly more time looking at the focus of a contraction flow than at the focus of an expansion flow, whereas children aged ≥ 5 years (with the exception of 8-year-olds) and adults spent significantly more time looking at the focus of an expansion flow than at the focus of a contraction flow. These results suggest that the expansion bias in time looking at the focus of a radial flow pattern typically observed among adults (Niemann et al., 1996; Shirai & Imura, 2016) emerges after a few years of age. In contrast, a longer time looking at the focus of contraction flow than at the focus of expansion flow was observed in 1-year-old children. This contraction bias was very similar to the results for younger infants (aged < 1 year) reported by Shirai and Imura (2016).

Additionally, post hoc analysis for the time looking at the AOI of the expansion flow among different age groups revealed that the looking time gradually increased between the relatively younger (i.e., 1- to 5-year-olds) and older (6- to 12-year-olds and adults) age groups (see Table 2a for details). The same set of analyses for the looking time at the AOI of the contraction flow also revealed a significant increase between the younger (1- to 6-year-olds) and older (10- to 12-year-olds and adults) groups (see Table 2a for details). These results indicate that mean looking time at the focus of both expansion and contraction flows increased gradually by approximately 5 or 6 years of age, and was comparable to the looking time exhibited by adults.

**Table 2:** The results of multiple comparisons for age differences in (a) time spent looking at the AOI, and (b) latency of the first gaze toward the AOI.

(a) Looking time to AOI													
	1-year-old	2-year-old	3-year-old	4-year-old	5-year-old	6-year-old	7-year-old	8-year-old	9-year-old	10-year-old	11-year-old	12-year-old	Adults
**Expansion**													
1-year-old		*p* = 1.000	*p* = 1.000	*p* = 1.000	*p* = 0.134	***p* < 0.001**	***p* < 0.001**	***p* = 0.001**	***p* < 0.001**	***p* < 0.001**	***p* < 0.001**	***p* < 0.001**	***p* < 0.001**
2-year-old			*p* = 1.000	*p* = 1.000	*p* = 0.356	***p* < 0.001**	***p* < 0.001**	***p* = 0.004**	***p* < 0.001**	***p* < 0.001**	***p* < 0.001**	***p* < 0.001**	***p* < 0.001**
3-year-old				*p* = 1.000	*p* = 1.000	***p* = 0.015**	*p* = 0.549	*p* = 1.000	*p* = 0.277	***p* < 0.001**	***p* = 0.002**	***p* = 0.026**	***p* < 0.001**
4-year-old					*p* = 1.000	***p* < 0.001**	***p* = 0.046**	*p* = 0.533	***p* = 0.020**	***p* < 0.001**	***p* < 0.001**	***p* = 0.001**	***p* < 0.001**
5-year-old						*p* = 0.184	*p* = 1.000	*p* = 1.000	*p* = 1.000	***p* = 0.005**	***p* = 0.025**	*p* = 0.290	***p* = 0.001**
6-year-old							*p* = 1.000	*p* = 1.000	*p* = 1.000	*p* = 1.000	*p* = 1.000	*p* = 1.000	*p* = 1.000
7-year-old								*p* = 1.000	*p* = 1.000	*p* = 1.000	*p* = 1.000	*p* = 1.000	*p* = 1.000
8-year-old									*p* = 1.000	*p* = 0.445	*p* = 1.000	*p* = 1.000	*p* = 0.153
9-year-old										*p* = 1.000	*p* = 1.000	*p* = 1.000	*p* = 1.000
10-year-old											*p* = 1.000	*p* = 1.000	*p* = 1.000
11-year-old												*p* = 1.000	*p* = 1.000
12-year-old													*p* = 1.000
**Contraction**													
1-year-old		*p* = 1.000	*p* = 1.000	*p* = 1.000	*p* = 1.000	*p* = 1.000	*p* = 1.000	*p* = 1.000	*p* = 1.000	***p* = 0.014**	*p* = 0.055	*p* = 0.230	***p* = 0.002**
2-year-old			*p* = 1.000	*p* = 1.000	*p* = 1.000	*p* = 1.000	*p* = 1.000	*p* = 0.806	*p* = 0.761	***p* < 0.001**	***p* = 0.002**	***p* = 0.011**	***p* < 0.001**
3-year-old				*p* = 1.000	*p* = 1.000	*p* = 1.000	*p* = 1.000	*p* = 1.000	*p* = 1.000	***p* = 0.005**	***p* = 0.020**	*p* = 0.093	***p* < 0.001**
4-year-old					*p* = 1.000	*p* = 1.000	*p* = 1.000	*p* = 0.935	*p* = 0.884	***p* < 0.001**	***p* = 0.003**	***p* = 0.014**	***p* < 0.001**
5-year-old						*p* = 1.000	*p* = 1.000	*p* = 1.000	*p* = 1.000	***p* = 0.005**	***p* = 0.023**	*p* = 0.104	***p* < 0.001**
6-year-old							*p* = 1.000	*p* = 1.000	*p* = 1.000	***p* = 0.049**	*p* = 0.175	*p* = 0.643	***p* = 0.007**
7-year-old								*p* = 1.000	*p* = 1.000	*p* = 1.000	*p* = 1.000	*p* = 1.000	*p* = 0.274
8-year-old									*p* = 1.000	*p* = 1.000	*p* = 1.000	*p* = 1.000	*p* = 0.795
9-year-old										*p* = 1.000	*p* = 1.000	*p* = 1.000	*p* = 0.842
10-year-old											*p* = 1.000	*p* = 1.000	*p* = 1.000
11-year-old												*p* = 1.000	*p* = 1.000
12-year-old													*p* = 1.000

(b) Latency of the first gaze toward AOI													
	1-year-old	2-year-old	3-year-old	4-year-old	5-year-old	6-year-old	7-year-old	8-year-old	9-year-old	10-year-old	11-year-old	12-year-old	Adults
**Expansion**													
1-year-old		*p* = 1.000	*p* = 1.000	*p* = 1.000	*p* = 1.000	*p* = 0.667	*p* = 0.074	***p* = 0.036**	*p* = 0.351	***p* = 0.007**	*p* = 0.134	***p* = 0.002**	***p* = 0.012**
2-year-old			*p* = 1.000	*p* = 1.000	*p* = 1.000	*p* = 1.000	*p* = 0.243	*p* = 0.126	*p* = 0.998	***p* = 0.027**	*p* = 0.417	***p* = 0.009**	***p* = 0.046**
3-year-old				*p* = 1.000	*p* = 1.000	*p* = 1.000	*p* = 0.495	*p* = 0.268	*p* = 1.000	*p* = 0.062	*p* = 0.821	***p* = 0.023**	*p* = 0.103
4-year-old					*p* = 0.766	***p* = 0.011**	***p* < 0.001**	***p* < 0.001**	***p* = 0.005**	***p* < 0.001**	***p* = 0.001**	***p* < 0.001**	***p* < 0.001**
5-year-old						*p* = 1.000	*p* = 1.000	*p* = 1.000	*p* = 1.000	*p* = 0.846	*p* = 1.000	*p* = 0.380	*p* = 1.000
6-year-old							*p* = 1.000	*p* = 1.000	*p* = 1.000	*p* = 1.000	*p* = 1.000	*p* = 1.000	*p* = 1.000
7-year-old								*p* = 1.000	*p* = 1.000	*p* = 1.000	*p* = 1.000	*p* = 1.000	*p* = 1.000
8-year-old									*p* = 1.000	*p* = 1.000	*p* = 1.000	*p* = 1.000	*p* = 1.000
9-year-old										*p* = 1.000	*p* = 1.000	*p* = 1.000	*p* = 1.000
10-year-old											*p* = 1.000	*p* = 1.000	*p* = 1.000
11-year-old												*p* = 1.000	*p* = 1.000
12-year-old													*p* = 1.000
**Contraction**													
1-year-old		*p* = 1.000	*p* = 1.000	*p* = 1.000	*p* = 1.000	*p* = 1.000	*p* = 1.000	*p* = 1.000	*p* = 1.000	*p* = 1.000	*p* = 1.000	*p* = 1.000	*p* = 1.000
2-year-old			*p* = 1.000	*p* = 1.000	*p* = 1.000	*p* = 1.000	*p* = 0.806	*p* = 0.191	*p* = 1.000	*p* = 0.128	*p* = 0.084	***p* = 0.018**	*p* = 0.062
3-year-old				*p* = 1.000	*p* = 1.000	*p* = 1.000	*p* = 1.000	*p* = 1.000	*p* = 1.000	*p* = 1.000	*p* = 1.000	*p* = 0.849	*p* = 1.000
4-year-old					*p* = 1.000	*p* = 1.000	*p* = 1.000	*p* = 1.000	*p* = 1.000	*p* = 1.000	*p* = 1.000	*p* = 0.701	*p* = 1.000
5-year-old						*p* = 1.000	*p* = 1.000	*p* = 0.653	*p* = 1.000	*p* = 0.457	*p* = 0.312	*p* = 0.077	*p* = 0.238
6-year-old							*p* = 1.000	*p* = 0.349	*p* = 1.000	*p* = 0.239	*p* = 0.159	***p* = 0.036**	*p* = 0.120
7-year-old								*p* = 1.000	*p* = 1.000	*p* = 1.000	*p* = 1.000	*p* = 1.000	*p* = 1.000
8-year-old									*p* = 1.000	*p* = 1.000	*p* = 1.000	*p* = 1.000	*p* = 1.000
9-year-old										*p* = 1.000	*p* = 1.000	*p* = 1.000	*p* = 1.000
10-year-old											*p* = 1.000	*p* = 1.000	*p* = 1.000
11-year-old												*p* = 1.000	*p* = 1.000
12-year-old													*p* = 1.000

Bold characters represent statistically significant differences (*p* < .05) among age groups. Because all age group combinations (78 pairs) were analyzed in each flow condition, the Bonferroni-corrected α (= 0.05/78) was used.

### Additional analysis of looking time (Fig. 3b,c)

The main findings from the looking time data were that the youngest children showed a contraction bias (less time looking at the focus of the expansion flow versus the focus of the contraction flow) while participants aged ≥ 5 years showed an expansion bias (more time spent looking at the focus of the expansion flow versus the focus of the contraction flow). Although these findings may represent developmental changes in gaze behavior regarding focus when viewing radial flow patterns, the findings may be explained by another factor: the amount of time spent time looking at the entire flow pattern may change with age. Thus, the observed developmental change in time spent looking at the AOI may simply reflect a change in looking behavior for the whole flow pattern. To test this, we performed additional analysis of the mean time spent looking at the whole area of the computer monitor (AOIW).

A three-way ANOVA with a linear mixed-effects model (fixed factors: age [13] × flow direction [2] × dot speed [2]; random factor: participants) conducted for the AOIW data revealed that the main effect of age and the two-way interaction (age × flow × speed) were statistically significant (*F*(12,247) = 3.830, *p* < 0.001, *η*_*p*_^2^ = 0.16 (95% CI: 0.05, 0.21); *F*(12,247) = 2.171, *p* = 0.014, *η*_*p*_^2^ = 0.10 (95% CI: 0.00, 0.13)). Although many different post hoc analyses could have been conducted for the two-way interaction, post hoc analyses of the simple interaction between age and flow direction in each the speed condition were useful to examine developmental differences in gaze patterns between the expansion/contraction flows at the AOIW (whole area of the monitor). Hence, we report the results of the post hoc analyses for the simple interaction between age and flow direction under each of the speed conditions as follows.

A two-way ANOVA with a linear mixed-effects model (fixed factors: age [13] × flow direction [2]; random factor: participants) revealed that only the main effect of age was statistically significant in the low-speed condition (*F*(12,247) = 4.090, *p* < 0.001, η_*p*_^2^ = 0.17 (95% CI: 0.06, 0.27)); the main effect of flow direction and interaction between age and flow direction were not statistically significant (*F*(1,247) = 1.506, *p* = 0.221, η_*p*_^2^ = 0.00 (95% CI: 0.00, 0.04); *F*(12,247) = 1.767, *p* = 0.054, η_*p*_^2^ = 0.08 (95% CI: 0.00, 0.14)). The lack of a significant simple main effect of flow direction or interaction effect indicates that there was no significant difference in looking time between the expansion and contraction flows in the low-speed condition. In contrast, two-way ANOVA with a linear mixed-effects model (fixed factors: age [13] × flow direction [2]; random factor: participants) revealed that the main effect of age and interaction between age and flow direction were statistically significant in the high-speed condition (*F*(12,247) = 2.820, *p* = 0.001, η_*p*_^2^ = 0.12 (95% CI: 0.02, 0.16); *F*(12,247) = 1.846, *p* = 0.041, η_*p*_^2^ = 0.08 (95% CI: 0.00, 0.15)). The main effect of flow direction was not statistically significant (*F*(1,247) = 0.683, *p* = 0.409, η_*p*_^2^ = 0.00 (95% CI: 0.00, 0.03)). Post hoc analyses revealed that the difference between the two flow directions in each age group was statistically significant (*p* < 0.05) only in 1-, 6-, and 7-year-olds under the high-speed condition.

These results indicate that the difference in overall pattern of looking time between the expansion and contraction flows was inconsistent between the AOI data (the focus of the radial flows) and AOIW data (the whole flow patterns). Most of the participants aged ≥ 5 years had a significantly different looking time between the flow patterns in the AOI analysis. In contrast, the AOIW analysis revealed no statistically significant difference in looking time between the expansion and contraction flows under the low-speed condition, and only a few age groups showed a significant difference in looking time between the expansion and contraction flows under the high-speed condition. Additionally, the direction of the bias in looking time for the expansion/contraction flows was partially inconsistent between the AOIW and AOI data. For instance, the 7-year-olds exhibited a contraction bias in the AOIW analysis under the high-speed condition (right panel in Fig. 3b), whereas they showed an expansion bias in the AOI analysis (right panel in Fig. 3a).

We next examined the difference in looking time between the expansion and contraction flows, as an index of expansion/contraction asymmetry in the AOIW analysis (Fig. 3c). To calculate the asymmetry index for each participant, we divided the total looking time for the expansion flow by the total looking time for both the expansion and contraction flows, separately for each speed condition. Mean index values higher and lower than 0.5 indicate looking behaviors biased toward expansion and contraction flows, respectively. Figure 3c shows the asymmetry index for the AOIW analysis. According to a Bonferroni-corrected (*α* = 0.05/26) two-tailed one-sample t-test, performed for all 26 (13 age groups × 2 speed conditions) mean indexes, no mean index value reached significance. Thus, there was no significant difference in looking time between the expansion and contraction flows in the AOIW analysis.

Taken together, the results of the AOIW analysis suggest that the developmental patterns found in the AOI data reflect specific gaze patterns toward the focus of radial flow patterns and do not reflect differences in fixation patterns for the screen as a whole.

### Summary of individual results for latency of the first gaze to AOI (Fig. 4)

**Figure 4:**
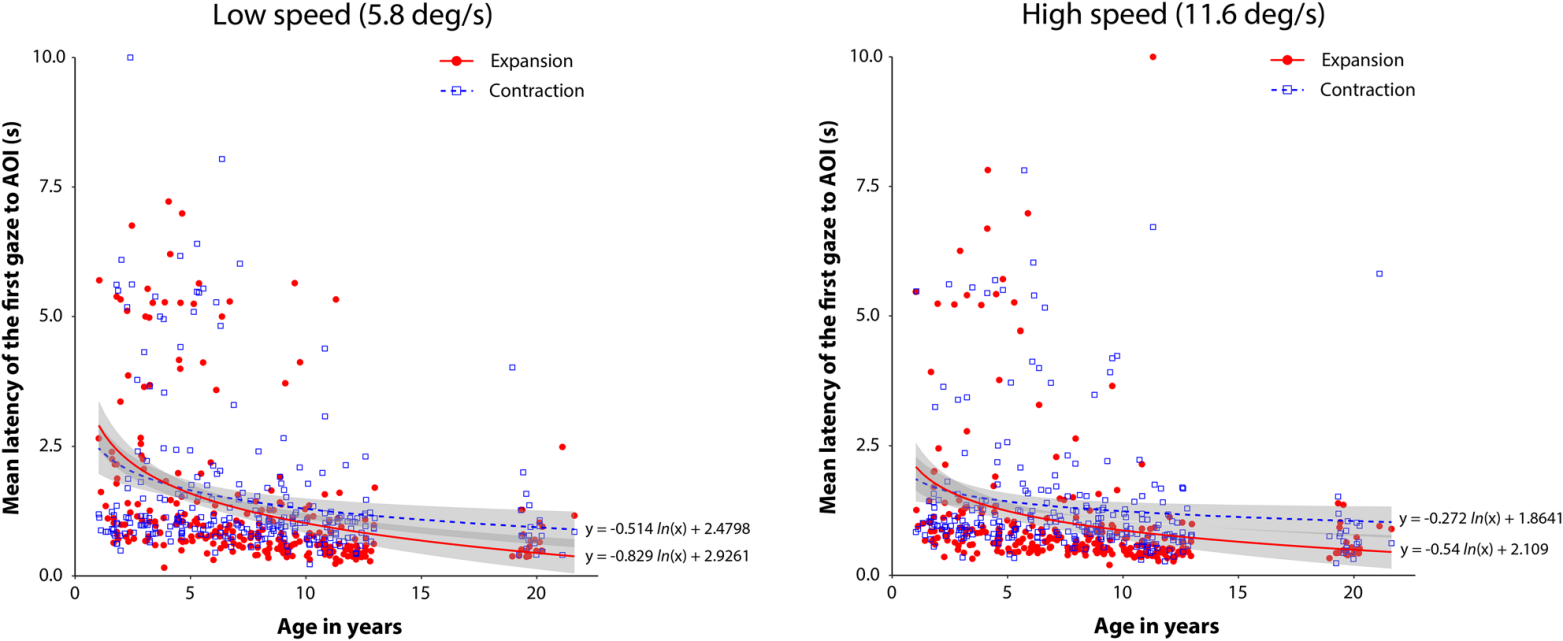
Individual mean latency of the first gaze to AOI under each experimental condition. The left and right panels summarize the results for the low- and high-speed conditions, respectively. In each panel, the vertical axis shows the mean latency of the first gaze to AOI, and the horizontal axis shows the ages of the individual participants in years. Red circles and blue squares indicate the individual mean latency for the expansion and contraction flow patterns, respectively. Solid red and dotted blue lines represent logarithmic regression curves for the expansion and contraction flow conditions, respectively. Gray stripes indicate 95% confidence intervals. The regression curves and confidence intervals were obtained using the ggplot2 R package (ver. 3.3.5).

Figure 4 shows the individual results for the mean latency of the first gaze to AOI for the experimental conditions. Overall, the results showed a modest decrement in the mean latency with age under both the low- (5.8 deg/s) and high-speed (11.6 deg/s) conditions, and the expansion bias, i.e., shorter latency to the AOI of expansion than contraction flows, seems to be less pronounced across the whole age range.

### Main analysis for latency (Fig. 5a)

**Figure 5:**
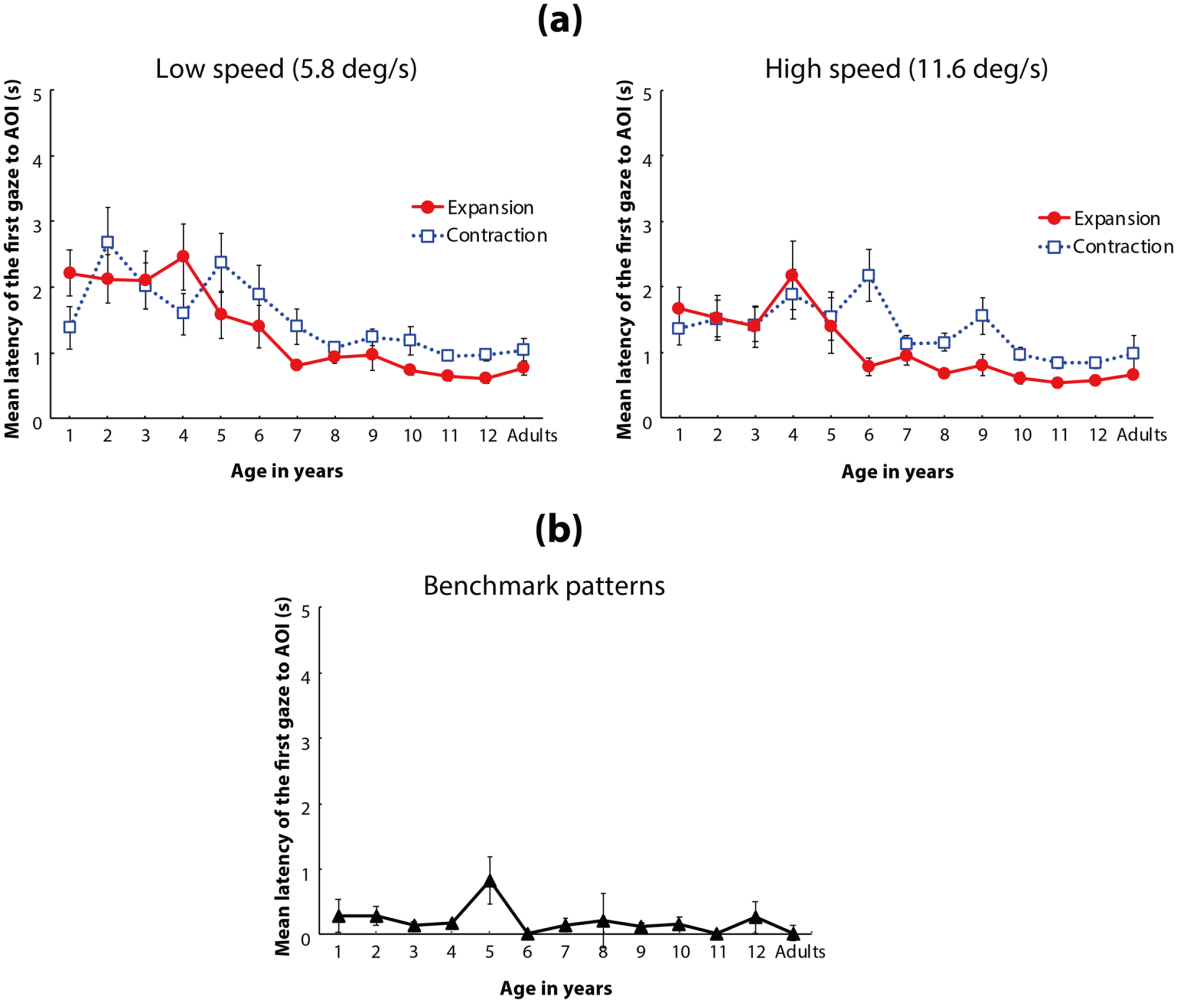
(a) Mean latency of the first gaze toward the AOI of the flow patterns. In each panel, the vertical axis shows the mean latency to the AOI, and the horizontal axis indicates the age group of the participants. Solid red lines with circles represent the mean latency for expansion flow patterns, and dotted blue lines with open squares represent the mean latency for contraction flows. Left and right panels indicate the results under low-speed (5.8°/s) and high-speed (11.6°/s) conditions, respectively. Error bars indicate ± 1 *SEM*. (b) Mean latency of the first gaze toward the AOI of the benchmark patterns. The vertical bar shows the mean latency to the AOI, and the horizontal bar indicates the age group of the participants. Error bars indicate ± 1 *SEM*.

A three-way ANOVA with a linear mixed-effects model (fixed factors: age [13] × flow direction [2] × dot speed [2]; random factor: participants) revealed that the main effects of age, flow direction, and dot speed were statistically significant (*F*(12,247) = 6.415, *p* < 0.001, η_*p*_^2^ = 0.24 (95% CI: 0.12, 0.30); *F*(1,247) = 6.941, *p* = 0.009*,* η_*p*_^2^ = 0.03 (95% CI: 0.00, 0.08); and *F*(1, 247) = 8.629, *p* = 0.004, η_*p*_^2^ = 0.03 (95% CI: 0.00, 0.09); respectively). The interaction between age and flow direction (*F*(12,247) = 2.189, *p* = 0.007*,* η_*p*_^2^ = 0.10 (95% CI: 0.00, 0.13)) was also statistically significant. The other interactions were not statistically significant (age × speed: *F*(12,247) = 1.180, *p* = 0.298*,* η_*p*_^2^ = 0.05 (95% CI: 0.00, 0.10); direction × speed: *F*(1,247) = 0.576, *p* = 0.448*,* η_*p*_^2^ = 0.00 (95% CI: 0.00, 0.03); age × direction × speed: *F*(12,247) = 0.825, *p* = 0.624*,* η_*p*_^2^ = 0.04 (95% CI: 0.00, 0.05)). As described above for the looking time data, the results regarding the interaction between age and flow direction are critical for discussing the development of asymmetric gaze responses toward the focus of a radial optic flow. Thus, we hereafter focus on the relevant analyses for the interaction between age and flow direction.

Post hoc comparisons revealed that the simple main effect of flow direction was statistically significant only in 1-, 4-, and 6-year-olds (*ps* < 0.05). These results indicated that the 1- and 4-year-olds showed significantly shorter latency to reach the focus of contraction, compared with expansion flow (contraction bias); in contrast, the 6-year-olds showed significantly shorter latency to reach the focus of expansion flow, compared with contraction flow (expansion bias). All other age groups (2, 3, and > 5 years, with the exception of 6-year-olds) showed no significant difference in latency data between the expansion and contraction flows, while they entirely showed shorter latency to the focus of expansion flow, compared with contraction flow. This significant contraction bias in generally younger individuals and non-significant expansion bias in generally older individuals are consistent with the findings reported by Shirai and Imura (2016).

Post hoc comparisons for the age differences in latency in the expansion flow revealed statistically significant differences between the relatively younger (i.e., 1- to 4-year-olds) and older (i.e., 6- to 12-year-olds and adults) age groups (Table 2b). On the other hand, multiple comparisons for the age difference in contraction flow revealed statistically significant differences in latency only between 2- and 12-year-olds and between 6- and 12-year-olds (Table 2b). These results indicate that mean latency to the focus of expansion flow decreased by 4 years of age, while the latency to the focus of contraction flow had a comparatively modest developmental change between infancy/toddlerhood and adulthood.

### Additional analysis for latency (Fig. 5b)

We compared the latencies to reach the participants’ gaze to the benchmark stimulus (a white small square moved along the same motion path as the focus of the radial flow in our stimuli) among the age groups to estimate the developmental change in the ability of gaze control itself. A one-way ANOVA with a linear mixed-effects model (fixed factor: age; random factor: participants) revealed that the main effect of age was not statistically significant (*F*(12,247) = 0.889, *p* = 0.559, η_*p*_^2^ = 0.04 (95% CI: 0.00, 0.05)). These results verify that the developmental changes observed in the latency data (latency for the participants’ gaze to reach the AOI decreased with age) did not originate from immature gaze control abilities of younger individuals but from children’s inability to detect the focus of radial flow patterns.

## General discussion

The looking time data indicated that although the gaze patterns toward the focus of radial expansion/contraction flows revealed a contraction bias (longer looking time for contraction, compared with expansion flows) in the youngest age group (1-year-olds); the contraction bias disappeared in older age groups. Furthermore, the expansion bias appears around (or after) 5 years of age. In the latency data, the mean latency to look at the focus of radial flows showed a contraction bias (shorter latency for contraction compared with expansion flows) in some younger groups (only 1- and 4-year-olds); the contraction bias disappeared in the later age ranges. These results suggest that although the contraction bias in gaze responses to the radial expansion/contraction flows is present during an earlier stage of life, the contraction bias disappears around the end of toddlerhood. Then, the gaze responses begin to change toward the expansion bias from early childhood. Moreover, our data also show that the ability to detect the focus of radial flow (taking expansion and contraction together) gradually develops through infancy to childhood; time looking at the focus of radial flows increased with age by 5–6 years of age, while latency to the focus of radial flows decreased with age during the same period. Taken together, the findings indicate that gaze responses toward the focus of radial flows, one possible visual cue to detect and control locomotor action, gradually change into adult-like form (i.e., an expansion bias in detection of the focus of radial optic flow that may be useful to detect and control locomotor direction) thorough early childhood.

Although numerous previous studies investigated developmental trends in the ability to detect radial optic flow from infancy to adulthood, the period in which remarkable developmental change in this ability was observed has varied considerably. Some previous studies reported that sensitivity to radial optic flow emerges in the first few months of life (e.g., visual preference^15–18^ or brain activity^19–21^) and that sensitivity to radial optic flow increases through the following year (e.g., brain activity^21^). Later in development, at about 4–8 years of age, adult-like brain responses to various optic flow patterns including radial expansion/contraction are observed^22^. These previous findings indicated that the ability to detect radial optic flow emerges very early in life and achieves adult-like form during the first few years of life. On the other hand, several other studies reported more protracted development of radial optic flow perception. For instance, the detection accuracy for global motion patterns, including radial flow, was significantly lower for children aged 5–8 years than adults^23^, the motion coherence threshold for the detection of radial optic flow patterns decreased from 6 to 16 years of age^24^, and estimation of the rigidity of a perceived flat plane from a radially expanding optic flow pattern showed adult-like form in 9- to 11-year-olds but not in 6- to 9-year-olds^25^. The current findings also support the conclusion that some aspects of the visual processing of radial optic flow patterns have a more protracted developmental time course than other aspects. Because radial optic flow seems to be related to a variety of important adaptive actions (e.g., guiding locomotion^1–5^, avoiding a collision^26–30^, compensating postural fluctuation^31,32^, and estimating geometrical structures of objects^33,34^), it is plausible that developmental trends in visual abilities related to radial optic flow detection/perception also vary considerably. Consideration of the relationship between adaptive actions and radial optic flow perception would be an important future task to understand the development of optic flow perception.

Notably, the contraction bias in the current looking time results for the youngest age group was apparently similar to previously reported contraction bias in brain activity (larger steady-state visual evoked potentials to contraction than expansion flow) in 4-month-old infants (Shirai et al. 2009)^20^. However, the contraction bias observed in the present study disappeared in the older (> 4 years) age groups, whereas the previously reported contraction bias in brain activity continued to adulthood^20^. This difference between the current and previous studies suggests that these two contraction biases must be related to distinct aspects of radial optic flow processing. The developmental change in the ability to detect the focus of radial flows and the transition from a contraction to an expansion bias may be driven by maturation of the ability to organize local visual components into a global visual structure. Previous studies have shown that the perception of global visual patterns substantially develops through (or beyond) toddlerhood/childhood^35–39^. Shirai and Imura (2016)^12^ suggested that the contraction bias observed in younger individuals may originate from comparatively immature global motion processing at those ages. For instance, young infants tend to shift their gaze in the direction of a moving visual pattern^40,41^. Such a tendency in gaze responses to motion patterns would make young infants more responsive to the direction of each dot than to a configural structure of a whole flow pattern. It should be noted that the direction of the local subset of moving dots in a radial flow pattern is always headed toward (or away from) the focus of a contraction (or an expansion) in the retinal coordinates. Thus, if a very young observer’s gaze were easily captured by a more local component than by the global structure of radial optic flow, the observers gaze patterns would show a contraction bias, and they would tend to shift their gaze toward the focus of a contraction flow having local motion components toward the focus. In contrast, during later developmental stages in childhood or older ages, the ability of global motion processing improves, and the ability to perceive a configurable structure of radial optic flow (including the focus of the flow pattern) also improves. Thus, older observers in those developmental stages may be more sensitive to the focus of radial flow and could shift their attention/gaze toward the focus of radial optic flow, which provides ecologically more important visual information, compared with local flow components.

Another important result of this study was that gaze responses to flow patterns appeared to have considerable individual variability (see Figs. 2 and 4). Under the current experimental conditions, if a participant’s gaze was precisely fixed at the center of the computer screen throughout an experimental trial, the total looking time and latency for a given trial should be 5 s and 1.25 s, respectively. These “chance” looking time and latency values were estimated as follows. The distance between the center of the screen (and the participant’s gaze) and the nearest edge of the AOI was approximately 4.6 deg (corresponding to 134 pixels on the monitor) at the start of each trial. The AOI (and thus the edge) moved toward the center of the screen at a rate of about 3.7 deg/s (corresponding to 107.2 pixels/s), so the points at the edge required 1.25 s (corresponding to the chance latency) to reach the center of the screen. After the edge reached the center of the screen, an AOI subtending 9.2 deg (corresponding to 268 pixels on the screen) would need 2.5 s to fully move away from the center of the screen. The AOI took 2.5 s to pass across the center of the screen during its return journey, such that the chance total looking time was 5 s. Some of the participants, particularly the younger ones, had a shorter looking time or longer latency for the AOI than the estimated chance values. This might be related to differences in visual saliency among the visual stimuli, which could be related to the movement rates of the flow patterns. The movement speed of the visual stimuli increased linearly toward the periphery; the higher dot speed at the periphery would likely be more salient and attract more visual attention compared to the radial flow (and thus the AOI), which had a relatively lower dot speed. This non-uniform distribution of visual saliency might influence the propensity to gaze toward the AOI and decrease the looking time (or increase the latency). Moreover, as mentioned in the previous paragraph, younger individuals may be more attracted to local features, such that the gaze would shift toward high-speed dots in peripheral areas more readily than toward low-speed dots in an AOI.

Additionally, locomotor action experience may play an important role in acquiring the expansion bias in gaze responses toward the focus of radial flow patterns. Locomotor behavior typically emerges around late infancy in humans, and changes into sophisticated forms during toddlerhood/childhood cf.^42^. Moreover, the amount of various physical activities, including locomotor actions, typically shows an inverted U-shape function from infancy to adolescence with a peak in childhood cf.^43^. The maturation of locomotor behaviors and the increase of physical activities around toddlerhood/childhood may result in increased opportunities to learn the ecological relationship between locomotor control and the focus of a radial optic flow pattern during those times. The development of global motion processing and the increase in locomotor experience in toddler/childhood are probably important factors to acquire the expansion bias and detect the focus of radial optic flow patterns. Because the current study did not directly analyze developmental changes in children’s daily locomotor experiences, this conclusion is speculative. However, future studies can empirically explore the relationship between developmental changes in locomotor experiences and the gaze behaviors to radial optic flows in individuals of various ages.
